# Supplementary material for: A model for microbial interactions and metabolomic alterations in Candida glabrata-Staphylococcus epidermidis dual-species biofilms
Source: PLoS One. 2022 Dec 13;17(12):e0279069. doi: 10.1371/journal.pone.0279069 (PMC9746963; doi:10.1371/journal.pone.0279069)
Supplement: Attachment — Submitted filename: Response to Reviewers.docx [file pone.0279069.s001.docx]

**Editor**

We are sorry it has taken so long to respond. There has been difficulty getting reviewers and several researchers as well as a proposed reviewer felt that the manuscript was asking a question about an interrelationship that does not physiologically exist. Additionally it was felt that C. glabrata is not a good biofilm maker. Indeed your studies really address dual species interaction and not necessarily in a biofilm. Consequently in the discussion you might include a few sentences on how your data would the interrelationship between the two species even if biofilms werent formed and why this would be important.

We wish to thank the academic editor for the comments and useful suggestions.

Reviewer 1 asks for more samples but I realize these experiments are usually only done with one strain - so you should comment in discussion that these results are for only 1 strain and differences may exist since it is known that the ability to make biofilms can be strain dependent.

According to the academic editor suggestion, text were added in the conclusion section to better explain the focus and relevance of our study which could be of wide general interest to the field of polymicrobial infections. Furthermore, as required by the editor, we also explicitly specified the strain dependence of our findings (see lines 835-845).

We have added text in the conclusion rather than in the discussion since we consider extension of our approach to the investigation of polymicrobial biofilm formation and growth to other strains and microbial species to be the object of future research.

**Reviewer 1**

This article entitled " Microbial Interactions and Metabolomic Alterations in Candida glabrata-Staphylococcus epidermidis Dual-Species Biofilms " is interesting and presents excellent results. The aim of the study is appropriate and novelty in polymicrobial infections field. The study is carried out in a very exhaustive way, both in the application of various techniques for the characterization of polymicrobial biofilms and in their discussion. This is an interesting, comprehensive and well-designed document, and I recommend it for publication.

We wish to thank the reviewer for the positive comments and useful suggestions to improve the manuscript.

However, I have some considerations to make:

Comments/Questions:
- The sample number is low, would it be possible to increase the number of isolates? Why, use the authors a clinical isolate of Staphylococcus epidermidis and a reference strain of Candida glabrata? More strains of C. glabrata and S. epidermidis should be used!

The focus of this manuscript is to understand if there was an interaction at biological and metabolomic level between *C. glabrata* and *S. epidermidis* in the polymicrobial environment. To achieve this goal we had, by necessity rather than by choice, to select a specified strain of each microorganism since, as all microbiological results, our findings may be strain dependent. This is in fact the reason why in Materials and Methods there is a precise information about the strains employed. In the revised version of the manuscript, we explicitly mentioned in the conclusion this aspect of our data and results. Comparing biological and metabolic characteristics of several strains of *C. glabrata* and *S. epidermidis* is outside of the scope of this paper although we consider it an excellent idea for future research.

- Line 182: "Sample preparation", the first two paragraphs are repetitive

As suggested by the reviewer, in the revised version of the manuscript a few sentences were merged to avoid repetitions and, furthermore, the paragraphs “Sample preparation” and “GC-MS analysis” were combined in only one paragraph named “Sample preparation and GC-MS analysis” in which the essential information concerning sample preparation, derivatization before GC-MS analysis, the instrumental method and the number of replicates were reported (see lines 183-206) .

- I think the data in table 2 would be more easily perceptible in graph.

As suggested by the reviewer, in the revised version of the manuscript Table 2 was replaced with a figure which was numbered as Figure 2 and, therefore, all subsequent figures and tables were renumbered.

- The quality of the imagens in figures should be improved.

As suggested by the reviewer, the quality and general aspect of all the figures were scrutinized and their quality greatly improved. A few images were restyled to make their content clearer. All images are provided in .tif format 300×300 pixel/in.
