# Supplementary material for: A model for microbial interactions and metabolomic alterations in Candida glabrata-Staphylococcus epidermidis dual-species biofilms
Source: PLoS One. 2022 Dec 13;17(12):e0279069. doi: 10.1371/journal.pone.0279069 (PMC9746963; doi:10.1371/journal.pone.0279069)
Supplement: Attachment — Submitted filename: Response to Reviewers.docx [file pone.0279069.s002.docx]

PONE-D-22-20604R1

A Model for Microbial Interactions and Metabolomic Alterations in Candida glabrata-Staphylococcus epidermidis Dual-Species Biofilms

PLOS ONE

**Academic Editor:** Marcos Pileggi, Ph.D

**Editor:** Authors should take into account the reviewers' observations, especially in relation to the conclusion drawn from experiments carried out only in microbiological media.

Answer: We thank the academic editor for considering our paper. We have carefully considered the Reviewer#2 observations concerning the use of a standard medium for growing single and dual species biofilms of *C.glabrata*/*S. epidermidis* biofilms. You can see our reply to this question in the Cover Letter and below in the answer to Reviewer#2.

**Reviewer#1: “**This article entitled " Microbial Interactions and Metabolomic Alterations in Candida glabrata-Staphylococcus epidermidis Dual-Species Biofilms " is interesting and presents excellent results. The aim of the study is appropriate and novelty in polymicrobial infections field. The study is carried out in a very exhaustive way, both in the application of various techniques for the characterization of polymicrobial biofilms and in their discussion. This is an interesting, comprehensive and well-designed document, and I recommend it for publication.”

Answer: We thank the Reviewer for the positive comments.

**Reviewer#2:** “The main drawback of this manuscript is about the conclusion drawn from the experiments carried out on just microbiological media. The authours claim that the choice of the strains are do to its prevalence in skin infections. So I strongly suggest that the metabolome study and gene regulation studies should be carried out using a simulated environment like a wound medium or a simulated wound infection model”

Answer: We thank the Reviewer for his/her observation which we have carefully considered.

We understand that the main concern of the Reviewer is on the use of a standard microbiological medium (TSB medium supplemented with 1% glucose) to grow single and dual-species biofilms of the targeted microorganisms which, according to the Reviewer, is the “main drawback” of the manuscript.

We respectfully observe that a standard microbiological medium is used in most of the literature dealing with the study of single species and polymicrobial biofilms of pathogenic microorganisms and this is a “choice” rather than a “drawback”. For instance, in the list of references in our paper there are not less than 10 papers which demonstrate this fact (see for instance Reference 31: [Huffines, J. T., & Scoffield, J. A. (2020). Disruption of Streptococcus mutans and Candida albicans synergy by a commensal streptococcus. *Scientific reports*, *10*(1), 1-10)].

Obviously, the medium can be enriched with components (biological fluids or tissue fragments, etc.) whose function is to simulate the biochemical environment of specific infected tissues or medical devices.

Both approaches are legitimate, and the choice depends on the objectives pursued.

In our manuscript we consider for the first time the biological and metabolic changes in the interaction of C. Glabrata-S. Epidermidis during biofilm formation, and the scope of our work is to collect information on fundamental biological and metabolic effects of this interaction. Although we have selected the microorganisms considering their role in real-world infections, it is perfectly reasonable to consider first their interaction when they are cultivated in vitro in a standard medium which contains only the ingredients necessary to ensure wellness of both microorganisms in order to trace a groove for future research.

Data collected under such conditions will not provide a definitive and universal model which can be applied without changes in all real-world situations but will give fundamental information which may be useful as a guide for further studies. In fact, in this field there is nothing like a universal model since any model is to some extend contingent and dependent on the data and conditions under which it was developed. The list of factors which may influence the model is very long and includes, among others, the strains of the microorganisms, the nature of the surface on which biofilms are deposited, the composition of the medium and the presence of exogenous substances which may or may not be present. This is because, in a specific work, factors which may influence the outcome of experiments must be accurately specified and controlled (as we did in our work).

In other words, each model represents only one tooth of a gear and we do not pretend that our work represents the whole gear, but we do pretend that it represents a single tooth of the gear.

The Reviewer suggestion of carrying out experiments using an environment simulating real-world infections is welcome since it is exactly what we are going to do in the continuing work, but it is, by definition, outside of the scope of the present work.

In any case we have accurately scrutinized the manuscript and made changes and added text and references to better specify its significance and further support our conclusions based on the presented data.

- We have modified the title to “**A Model for** Microbial Interactions and Metabolomic Alterations in *Candida glabrata*-*Staphylococcus epidermidis* Dual-Species Biofilms” which makes evident that the outcome of our paper is a basic model for the interactions between *C. glabrata*/*S. epidermidis* biofilms.

-By pursuing the same objective, we have substantially modified the “Introduction” and added text to better delineate the ambit and significance of our results.

- To better support our conclusions and to better connect them to previous studies, we have added further references and added text in the “Results” sections.

-We have also modified the “Conclusions” to make the significance of our work clearer.
